# Comparative study of *in situ* hybridization, immunohistochemistry and parasitological culture for the diagnosis of canine leishmaniosis

**DOI:** 10.1186/s13071-015-1224-4

**Published:** 2015-12-02

**Authors:** Marina C. Furtado, Rodrigo C. Menezes, Matti Kiupel, Maria F. Madeira, Raquel V. C. Oliveira, Ingeborg M. Langohr, Fabiano B. Figueiredo

**Affiliations:** Laboratório de Pesquisa Clínica em Dermatozoonoses em Animais Domésticos, Instituto Nacional de Infectologia Evandro Chagas, Fundação Oswaldo Cruz, Rio de Janeiro, RJ Brazil; Department of Pathobiology and Diagnostic Investigation, College of Veterinary Medicine, Michigan State University, Lansing, MI USA; Laboratório de Vigilância em Leishmanioses, Instituto Nacional de Infectologia Evandro Chagas, Fundação Oswaldo Cruz, Rio de Janeiro, RJ Brazil; Laboratório de Epidemiologia Clínica, Instituto Nacional de Infectologia Evandro Chagas, Fundação Oswaldo Cruz, Rio de Janeiro, RJ Brazil; Department of Pathobiological Sciences, School of Veterinary Medicine, Louisiana State University, Baton Rouge, LA USA

**Keywords:** *Leishmania infantum*, Dog, Diagnosis, *In situ* hybridization, Immunohistochemistry, Parasitological culture

## Abstract

**Background:**

The establishment of an accurate diagnostic protocol for canine visceral leishmaniosis (CanL) is a significant laboratory challenge and the lack of a reliable reference standard is one of the major problems. The aim of this study was to compare *in situ* hybridization (ISH), immunohistochemistry (IHC) and parasitological culture (PC) for detection of *L. infantum* in skin, spleen, lymph node and bone marrow of clinically healthy and sick seropositive dogs.

**Findings:**

The study included 65 dogs positive with both DPP® and ELISA for anti-*Leishmania* antibodies. *In situ* hybridization of spleen or lymph node had the highest positivity rates of *L. infantum* detection. The total positivity rates for IHC, ISH and PC were 70 %, 68.1 % and 65.8 %, respectively. When combining techniques, the positivity rates were 81.5 % in the spleen, 79.0 % in lymph nodes, 59.0 % in bone marrow and 52.3 % in the skin. The highest percentage of infected dogs (87.7 %) was detected by using lymph node samples. When examining only skin, positivity was significantly higher in sick dogs than in the clinically healthy dogs. Infection with *L. infantum* was confirmed in 95.8 % of sick dogs and in 82.4 % of healthy dogs.

**Conclusions:**

Considering the advantages of accurately diagnosing different *Leishmania* species and of being more sensitive than PC, ISH should be considered as reference standard test for the diagnosis of CanL. Spleen and lymph node are the most suitable tissues to confirm infection with *L. infantum* in seropositive dogs. The testing of only skin from clinically healthy dogs may result in a high percentage of false negative results.

## Background

Leishmanioses are caused by protozoan parasites of the *Leishmania* genus, that can be transmitted by phlebotomine sand flies to humans, domestic and wild mammals [1]. *Leishmania infantum* (syn *Leishmania chagasi*) is the etiological agent of zoonotic visceral leishmaniosis, for which the domestic dog represents the main reservoir in an urban environment [1].

Enzyme-linked immunosorbent assay (ELISA), indirect immunofluorescent antibody test (IFAT) and immunochromatographic rapid test *Dual Path Platform* (DPP®) are used to detect anti-*Leishmania* antibodies in dogs [2]. Serological assays are usually applied as screening tests because of their simple execution and rapid results, although, their accuracy for detecting canine visceral leishmaniosis (CanL) is limited [2]. Parasitological culture (PC) is considered the reference standard test, detecting *L. infantum* in 62.1 % to 82.2 % of seropositive dogs [3, 4]. However, PC is time consuming and can be impaired by microbiological contamination [5]. Polymerase chain reaction (PCR) has also been used for detecting parasitic DNA in tissue samples [6]. While PCR is a valuable tool for CanL diagnosis, it does not detect viable organisms and false positive results may occur due to laboratory contamination, while false negative results can be caused by the presence of inhibitory substances [7]. In contrast, histological tests show the presence of the parasite within lesions and allow confirmation of active infection in routinely formalin fixed tissues in a safe and timely manner [7]. Immunohistochemistry (IHC) is routinely performed to detect *Leishmania* in tissue sections, providing more sensitivity than conventional histopathology [8–10]. *In situ* hybridization (ISH) using a generic [11] or a specific probe [10] has been described for the diagnosis of *L. infantum* infection in dogs. The specific probe had a higher sensitivity than the generic probe, IHC or conventional histopathology in skin samples [10]. Skin (SK), spleen (SP), lymph node (LN) and bone marrow (BM) are the tissues most commonly collected for detection of *L. infantum* in dogs, but findings regarding their sensitivity are divergent [3, 4, 12–16].

The definition of an accurate diagnostic protocol for CanL is a significant laboratory challenge and the lack of a reliable reference standard is one of the major problems. The aim of this study was to compare the positivity rate of ISH versus IHC and PC for detection of *L. infantum* in SK, SP, LN and BM of clinically healthy and sick seropositive dogs.

## Methods

The study population included 65 dogs identified during a serological survey performed from 2011 to 2013 in the city of Barra Mansa, state of Rio de Janeiro, Brazil. All dogs were positive in both DPP® and ELISA tests for anti-*Leishmania* antibodies (DPP® CVL rapid test, BioManguinhos, Rio de Janeiro, Brazil and ELISA EIE® BioManguinhos, Rio de Janeiro, Brazil). Weight loss, alopecia, skin ulcer or nodule, exfoliative dermatitis, onychogryphosis, lymph node enlargement, splenomegaly, pale mucous membranes and skeletal muscle atrophy were considered clinical signs consistent with CanL [17]. After euthanasia, macroscopically intact SK from the scapular region, SP, popliteal LN and sternal BM were sampled for PC and the *Leishmania* isolates were identified by multi-locus enzyme electrophoresis [5]. For IHC and ISH, sections of SK, SP and LN and the clot of BM aspirate were fixed in 10 % buffered formalin and processed as for routine paraffin embedding. The IHC was performed using an in-house rabbit polyclonal anti*-Leishmania* serum [9]. For ISH, we used a specific antisense oligonucleotide probe as previously described [10].

Statistical analysis was performed using the Statistical Package for Social Sciences (SPSS) software for Windows (version 16.0). Infection with *L. infantum* was considered as confirmed when the parasite could be detected with at least one direct diagnostic test (PC, IHC or ISH). Fisher’s exact test was used to associate positivity rates in the various tissues with the clinical status. Differences were considered significant when *p* <0.05.

### Ethical approval

This study was approved by the Ethics Committee on Animal Use (LW-54/13-CEUA-FIOCRUZ).

## Results and discussion

**Fig. 1:**
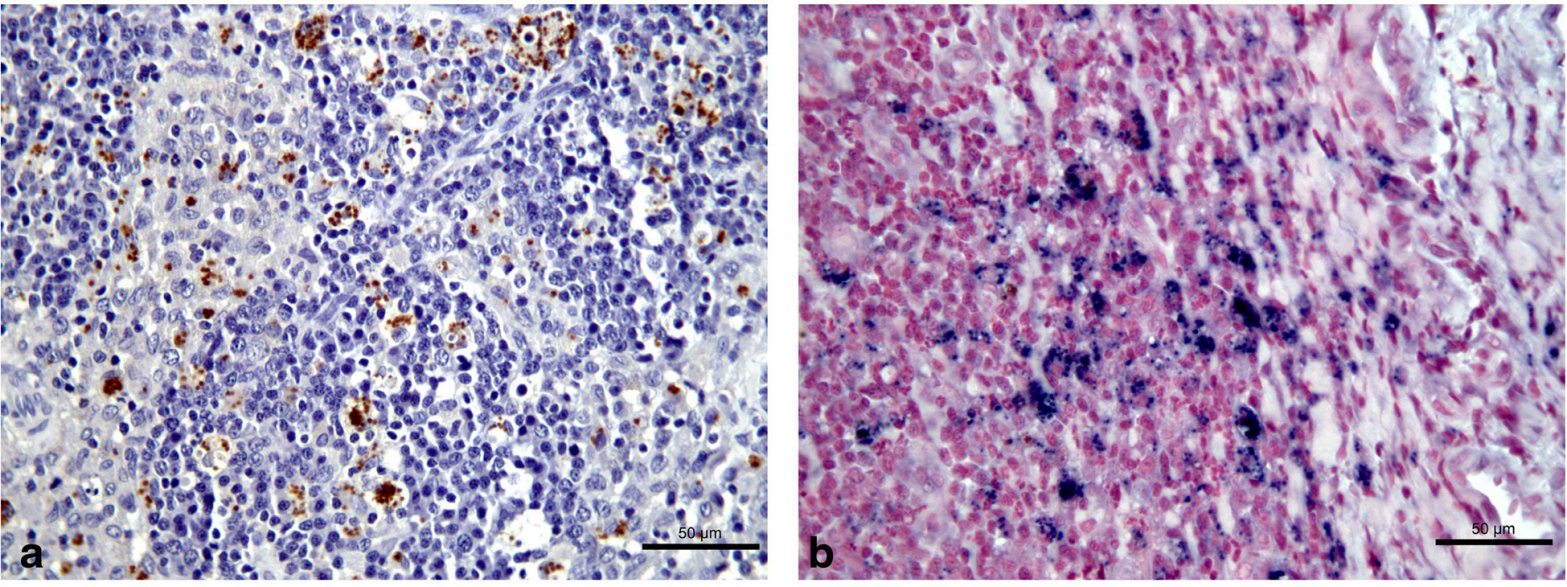
Immunohistochemistry and *in situ* hybridization on lymph node of a dog infected with *Leishmania infantum.* **a** IHC: dark brown stained *Leishmania* amastigote forms within macrophages. **b** ISH: dark blue labeled *Leishmania infantum* amastigote forms within macrophages

The positivity rates of *Leishmania* detection by IHC (Fig. 1a), ISH (Fig. 1b) and PC in SK, SP, LN and BM are listed in the Table 1. The total agreement was 88 % between ISH and IHC, 77 % between ISH and PC, and 75 % between IHC and PC. The percentages of clinically healthy as well as sick seropositive dogs with active *L. infantum* infection in different tissues are presented in the Table 2.

**Table 1:** Positivity rates of various diagnostic tests to confirm *Leishmania infantum* infection in 65 dogs

Techniques	Skin (*n* = 65)	Spleen (*n* = 65)	Lymph node (*n* = 65)	Bone marrow (*n* = 65)	Total (*n* = 260)
PC	37 (56.9 %)	52 (80.0 %)	47 (72.3 %)	35 (53.8 %)	171 (65.8 %)
IHC	34 (52.3 %)	53 (81.5 %)	53 (81.5 %)	42 (64.6 %)	182 (70.0 %)
ISH	31 (47.7 %)	54 (83.1 %)	54 (83.1 %)	38 (58.5 %)	177 (68.1 %)
Total (*n* = 195)	102 (52.3 %)	159 (81.5 %)	154 (79.0 %)	115 (59.0 %)	530 (67.9 %)

*PC* parasitological culture, *IHC* immunohistochemistry, *ISH in situ* hybridization, *n* number of examined samples

**Table 2:** Clinically healthy and sick seropositive dogs with confirmed *Leishmania infantum* infection in various tissues

	Healthy (*n* = 17)	Sick (*n* = 48)	Total (*N* = 65)	*p* value
Skin	8 (47.1 %)	39 (81.3 %)	47 (72.3 %)	0.011
Spleen	14 (82.4 %)	42 (87.5 %)	56 (86.2 %)	0.687
Lymph node	14 (82.4 %)	43 (89.6 %)	57 (87.7 %)	0.421
Bone marrow	14 (82.4 %)	40 (83.3 %)	54 (83.1 %)	1.000
Confirmed infection^a^	14 (82.4 %)	46 (95.8 %)	60 (92.3 %)	0.107

^a^Detection of *Leishmania* in at least one type of tissue

*p* <0.05

*In situ* hybridization applied to SP or LN sections had the highest positivity rates of *L. infantum* detection. However, regarding the total tests performed for the four types of tissue, IHC had the highest positivity rate. Since the polyclonal antibody used for IHC is not specific for *L. infantum* species and may cross-react with fungal antigens [9], false positive results may occur. In our study, no dog was positive using only IHC testing. Furthermore, all *Leishmania* isolates by PC were characterized as *L. infantum*. These findings confirm that the IHC technique did not detect other *Leishmania* species or infectious agents that could confound our results.

One dog was negative by PC in the four tested tissues, but positive by IHC and ISH. In this case, ISH was the only technique that could identify the species of *Leishmania* due to the use of a specific probe. The positivity rate of *Leishmania* detection by PC was lower than by IHC or ISH. The use of PC as reference standard in CanL diagnosis is questionable because contamination or poor adaption of the parasite to the medium may impair the sensitivity of this technique and underestimate the accuracy of other tests.

Our findings suggest that SP and peripheral LN are the most suitable tissues for detection of *L. infantum* in dogs. When all test results were compared, the SP had the highest positivity rate, but LN analysis identified the highest number of infected dogs. The inferior rates of detection of *L. infantum* in SK and BM may be due to the lower frequency of active infection in these tissues, a possibly lower parasite load when compared to LN and SP, or the stage of infection, which was not evaluated in this study. Both LN [4, 6, 14, 15] and SP [13, 16] have been recommended as the most suitable tissues for the diagnosis of *L. infantum* infection in dogs. Obtaining a LN biopsy is generally considered more practical and clinically safe than obtaining a SP biopsy and some professionals avoid SP sampling from live animals because of the invasiveness of the sampling technique and the risk of hemorrhage; however, a study evaluating the safety of SP aspirations in dogs, concluded that this procedure was effective and safe for the diagnosis of *L. infantum* infection [18].

In the present study, the testing of SK was sensitive for confirming infection in sick dogs only. Although SK is considered a suitable sample to detect *L. infantum* in dogs [12, 19], it should not be used in surveillance testing when the population includes clinically healthy dogs. Similarly to the sick dogs, the clinically healthy seropositive dogs had a high frequency of active *L. infantum* infection in SP, LN and BM, but a significant lower frequency in the SK. In a cross-sectional study, we cannot say whether the absence of clinical signs of CanL and SK parasitism were transitory or whether these findings are resistance characteristics. Nevertheless, as the parasite load in the SK of dogs is an indirect marker of infectiousness to the vector [20], we can assume that 53 % of the clinically healthy dogs did not play a role in the transmission of *L. infantum* at the time of the sample collection.

Five dogs included in this study had negative results in all confirmatory tests performed. Although they were serologically positive in two different tests, the possibility of false positive results exists, as serological assays for the diagnosis of *L. infantum* infection can cross-react with other infectious agents [21, 22]. It is also reasonable to classify these dogs as “exposed”, according to the criteria by Paltrinieri *et al.* [23], since they lived in endemic regions where *L. infantum* circulation had been confirmed. Dogs that have been exposed to *L. infantum,* but present no clinical signs of CanL and no evidence of active infection based on parasitological tests, may have anti-*Leishmania* antibodies [23].

The visceral leishmaniosis control program in Rio de Janeiro used to apply ELISA test followed by IFAT to identify dogs infected with *L. infantum*, but studies have shown that this protocol was inaccurate [24, 25]. Our results suggest that the protocol using DPP® and ELISA has a satisfactory positive predictive value, contributing to the decrease of euthanasia of non-infected dogs due to false positive results. However, in order to properly evaluate the accuracy of this screening protocol, a multicentric and carefully designed validation study is required.

In conclusion, *in situ* hybridization is a valuable test for the definite diagnosis of active *L. infantum* infection in dogs. Considering the advantages of being able to differentiate *Leishmania* species in tissue samples and being more sensitive than PC, ISH should be evaluated as the future reference standard test for CanL. Spleen and LN are the most suitable tissues to confirm infection with *L. infantum* in seropositive dogs, while SK should not be used as the only sample in clinically healthy dogs.

## Abbreviations

CanL: Canine visceral leishmaniosis
PC: Parasitological culture
IHC: Immunohistochemistry
ISH: *In situ* hybridization
SK: Skin
SP: Spleen
LN: Lymph node
BM: Bone marrow
ELISA: Enzyme-linked immunosorbent assay
IFAT: Indirect immunofluorescent antibody test
DPP®: *Dual path platform*
